# Supplementary material for: Low cost audiovisual playback and recording triggered by radio frequency identification using Raspberry Pi
Source: PeerJ. 2015 Apr 7;3:e877. doi: 10.7717/peerj.877 (PMC4393811; doi:10.7717/peerj.877)
Supplement: Supplemental Information 4 [file peerj-03-877-s004.docx]

As the author of the manuscrpit PeerJ 3605 testify that the person in Figure 2 (Alice Domalik, Dept. of Biology, Queen’s University, [9add2@queensu.ca](mailto:9add2@queensu.ca)) provided written permission on the 9th of February, 2015 to use the picture in publication in PeerJ. I am the copyright holder of the picture.
